# Regarding human cytomegalovirus in neuroblastoma

**DOI:** 10.1002/cam4.243

**Published:** 2014-04-17

**Authors:** Ola Forslund, Linda Holmquist Mengelbier, David Gisselsson

**Affiliations:** 1 Medical Microbiology, Department of Laboratory Medicine Malmö, Lund UniversityMalmö, Sweden; 2 Clinical Microbiology, Skåne Regional and University LaboratoriesMalmö, Sweden; 3 Division of Clinical Genetics, Department of Laboratory Medicine, Lund UniversityLund, Sweden; 4 Department of Pathology, Skåne Regional and University LaboratoriesLund, Sweden

Dear Sir/Madam,

Baryawno et al. have reported HCMV infection in medulloblastoma, the most frequent malignant pediatric brain tumor 1. Prompted by this, we recently investigated 12 cases of the childhood tumor neuroblastoma, all of which were found to be negative for HCMV by polymerase chain reaction (PCR) using primers for pp65 and by high throughput sequencing of five tumors 2. Neither could we detect pp65 or HCMV immediate-early (IE) proteins by immunohistochemistry in neuroblastoma cells 2. Recently, however, Wolmer-Solberg et al., reported that six human neuroblastoma cell lines and the vast majority of clinical neuroblastoma samples contained HCMV DNA and expressed HCMV proteins 3.

In order to address these discrepancies, we tested four of the six neuroblastoma cell lines used by Wolmer-Solberg for the presence of HCMV DNA. In addition we tested three clinical neuroblastoma samples (Table1). From the cell lines, DNA was extracted with the Total NA-kit (Roche, Stockholm, Sweden) using MagNA Pure LC (200 *μ*L input and 100 *μ*L output). DNA from clinical neuroblastoma samples was extracted with DNeasy Blood & Tissue Kit (Qiagen, Hamburg Germany). DNA quality for PCR was assessed in a separate assay by testing for the human beta-globin gene with a real-time PCR 4. For detection of HCMV DNA, we used real-time PCR with primers and probes for HCMV IE (forward 5′-CTCAACATAGTCTGCAGGAACGT, reverse 5′-GTGACCCATGTGCTTATGACTCTAT, FAM-TTGGTCACGGGTGTCTC-MGB), pp150 (forward 5′-CCGTGGGCGACAAAACG, reverse 5′-GGCGCGGGAACCTCTT, FAM-CAGCCGTCAGCCTCG-MGB) and UL55/gB (forward 5′-CGCCAACGGCCTTTCC, reverse 5′-GCTACCGCCCTACCTCAAG, FAM-CCCAGGCCGCTCATG-MGB) as specified in the supplementary section of the study from Wolmer-Solberg and colleagues 3. Primers and probes were ordered from DNA Technology (Risskov, Denmark) and Life Technologies (Stockholm, Sweden), respectively. We performed real-time PCR in 25 *μ*L containing 1× TaqMan Universal PCR master mixture (Applied Biosystems, Stockholm, Sweden), 0.5 *μ*mol/L of each primer, 0.1 *μ*mol/L probe, and 5 *μ*L sample. The PCR was carried out in an automated thermocycler (ABI 7500) as follows; 15′ at 95°C and then 45 cycles of 15″ at 95°C and 1′ at 57°C. The step at 57°C was used due to approximate Tm of primers calculated by Primer Express software 3.0 (Applied Biosystems). Limit of detection was determined from serial dilutions of 100,000 to 1 copies per PCR of HCMV DNA in a background of 50 ng human placenta DNA (art. D 7011, Sigma-Aldrich, Stockholm, Sweden). The HCMV DNA copy number of a stock solution of extracted HCMV DNA (AD169), used to prepare dilution series to determine limit of detection and to prepare positive control for the HCMV-DNA PCR, was determined by an automatic device (The COBAS® Ampliprep/COBAS® TaqMan® HCMV test, Roche). For testing specificity of the HCMV PCRs, we performed PCR with triplicate samples containing 50 ng/PCR of DNA from the SiHa cervical cell line, human DNA (Sigma-Aldrich, art. D 7011) as well as water controls.

**Table 1:** HCMV DNA was not detectable in human neuroblastoma cell lines or clinical neuroblastoma samples by the use of real-time PCR with primers and probes for three targets of the HCMV-genome.

Cell lines/clinical samples	Input DNA/25 μL PCR (ng)	HCMV-DNA PCR IE	HCMV-DNA PCR pp150	HCMV-DNA PCR UL55/gB	Sample adequacy Beta-globin PCR, (Ct)
SH-SY5Y	50	–	–	–	+ (24.9)
SK-N-BE(2)C	50	–	–	–	+ (26.0)
SK-N-AS	50	–	–	–	+ (24.3)
IMR-32	50	–	–	–	+ (24.3)
Sample 1	58	–	–	–	+ (26.5)
Sample 2	53	–	–	–	+ (25.5)
Sample 3	60	–	–	–	+ (25.9)

**Figure 1:**
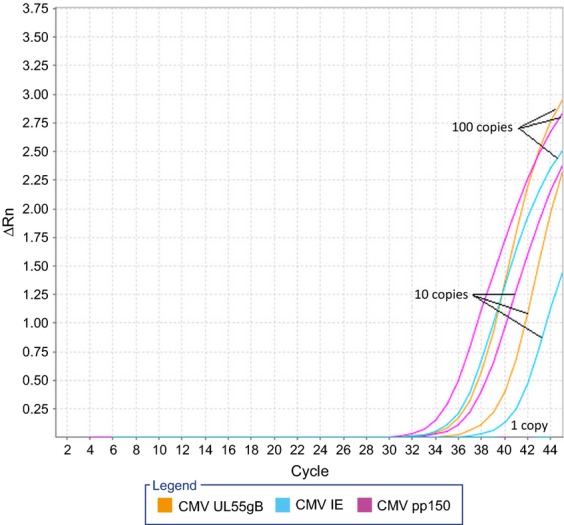
Limit of detection for HCMV was 10 copies/real-time PCR for each target gene IE(Ct: 40.9), pp150 (Ct: 37.9) and UL55/gB (Ct: 40.6). Cycle threshold (Ct) was automatically calculated by the ABI 7500 software v2.0.6. Input of 100, 10 and 1 HCMV copies are shown.

**Figure 2:**
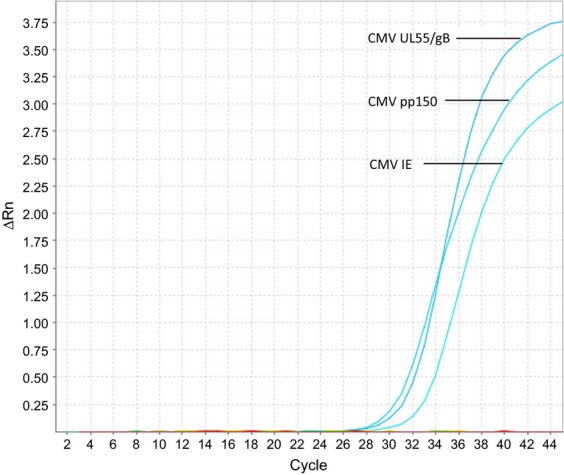
Neuroblastoma cell lines in triplicate (SH-SY5Y, SK-N-BE(2)C, SK-N-AS and IMR-32) were all negative for HCMV-DNA. Positive controls of 1000 HCMV-copies/real-time PCR generated expected curves for each target gene IE (Ct: 33.9), pp150 (Ct: 32.0) and UL55/gB (Ct: 32.9). Cycle threshold (Ct) was automatically calculated by the ABI 7500 software v2.0.6.

Each of the three HCMV PCR primer combinations showed satisfactory performance. The limit of detection was 10 copies/PCR for each of the targets IE, pp150 and UL55/gB (Fig.1). Identical limit of detection was demonstrated by the use of annealing step at 60°C for the PCRs (data not shown). No detection of HCMV DNA was observed in the SiHa cervical cell line, human DNA or water controls, indicating sufficient specificity of the PCRs (data not shown). DNA extracts from each of the neuroblastoma cell lines and clinical samples were tested in triplicate by each of the three primer combinations. Input of 50–60 ng DNA, corresponding to about 7500 to 9000 cells, was added to each PCR. No detection of HCMV DNA was observed in any of the samples (Table1 and Fig.2). Thus, we could not replicate the data from the recently published report by Wolmer-Solberg and colleagues 3.

Based on our previous study 2 and the present results, we remain skeptical towards the prevalence of HCMV DNA in a majority of neuroblastomas.
